# Striatal Beta Oscillation and Neuronal Activity in the Primate Caudate Nucleus Differentially Represent Valence and Arousal Under Approach-Avoidance Conflict

**DOI:** 10.3389/fnins.2020.00089

**Published:** 2020-02-07

**Authors:** Ken-ichi Amemori, Satoko Amemori, Daniel J. Gibson, Ann M. Graybiel

**Affiliations:** ^1^The Hakubi Center for Advanced Research and Primate Research Institute, Kyoto University, Inuyama, Japan; ^2^McGovern Institute for Brain Research and Department of Brain and Cognitive Sciences, Massachusetts Institute of Technology, Cambridge, MA, United States

**Keywords:** caudate nucleus, beta oscillation, valence, arousal, approach–avoidance conflict, cognitive engagement, decision-making, primate

## Abstract

An approach-avoidance (Ap–Av) conflict arises when an individual has to decide whether to accept or reject a compound offer that has features indicating both reward and punishment. During value judgments of likes and dislikes, arousal responses simultaneously emerge and influence reaction times and the frequency of behavioral errors. In Ap–Av decision-making, reward and punishment differentially influence valence and arousal, allowing us to dissociate their neural processing. The primate caudate nucleus (CN) has been implicated in affective judgment, but it is still unclear how neural responses in the CN represent decision-related variables underlying choice. To address this issue, we recorded spikes and local field potentials (LFPs) from the CN while macaque monkeys performed an Ap–Av decision-making task. We analyzed 450 neuronal units and 667 beta oscillatory activities recorded during the performance of the task. To examine how these activities represented valence, we focused on beta-band responses and unit activities that encoded the chosen value (ChV) of the compound offer as derived from an econometric model. Unit activities exhibited either positive (65.0% = 26/40) or negative (35.0% = 14/40) correlations with the ChV, whereas beta responses exhibited almost exclusively positive correlations with the ChV (98.4% = 62/63). We examined arousal representation by focusing on beta responses and unit activities that encoded the frequency of omission errors (FOE), which were negatively correlated with arousal. The unit activities were either positively (65.3% = 17/26) or negatively (34.6% = 9/26) correlated with the FOE, whereas the beta responses were almost entirely positively correlated with the FOE (95.8% = 23/24). We found that the temporal onset of the beta-band responses occurred sequentially across conditions: first, the negative-value, then low-arousal, and finally, high-value conditions. These findings suggest the distinctive roles of CN beta oscillations that were sequentially activated for the valence and arousal conditions. By identifying dissociable groups of CN beta-band activity responding in relation to valence and arousal, we demonstrate that the beta responses mainly exhibited selective activation for the high-valence and low-arousal conditions, whereas the unit activities simultaneously recorded in the same experiments responded to chosen value and other features of decision-making under approach-avoidance conflict.

## Introduction

The classic work of Miller (1944) introduced researches on the conflict that is felt when individuals must decide whether to accept or to reject something that has attractive and unpleasant qualities and what rewarding or negative results they would receive would depend on their decision. This conflict situation was instantiated in the so-called approach-avoidance (Ap–Av) conflict task, which, with modifications, has been used to study the reactions of humans, old world monkeys, rats, and mice (Miller, 1944; Elliot, 2008; Aupperle and Paulus, 2010; Amemori and Graybiel, 2012; McNaughton and Corr, 2014; Friedman et al., 2015, 2017; Amemori et al., 2018; Ironside et al., 2019). In such tasks, arousal responses occur, and these can be estimated by examining reaction times (RT) and the frequency of omission errors (FOE) (Roesch and Olson, 2004; Amemori et al., 2015). The rewarding and punishing aspects of the offers, that is, their valence can be distinguished from arousal, allowing one the chance to dissociate the biological basis of these different behavioral responses (Lang et al., 1998). For instance, a rewarding offer increases both the valence and the level of arousal. Contrarily, a punishment offer reduces the valence but activates the arousal level by enhancing the motivation to avoid it. The increase in the arousal level has been shown to be associated with changes in physiological measurements such as increases in pupil size and skin conductance (Loggia et al., 2011) and reductions of the RTs and the FOEs (Amemori et al., 2015). Because these autonomous nervous systems similarly responded to pleasant and unpleasant visual pictures (Bradley et al., 2008), researchers have considered it possible for arousal processes that respond to the saliency of such offers (Bradley et al., 1992) to exist in the nervous system (Matsumoto and Hikosaka, 2009).

Valence and arousal give us a two-process view that could underlie affective judgment. Psychological theories pointed out that sustained cognitive engagement is a necessary factor for the performance of affective judgments (Lazarus, 1991). Ap–Av decision-making could thus consist of multiple processes, including valence-related processes and those of cognitive engagement related to arousal. Researchers have identified distinctive groups of neuronal activities individually involved in either valence or arousal (Roesch and Olson, 2004; Colibazzi et al., 2010; Amemori et al., 2015; Ebitz and Platt, 2015). However, few studies have addressed the neuronal interaction between valence and arousal during Ap–Av decision-making in the primate striatum.

The involvement of striatal beta oscillation in movement preparation is well-documented (Murthy and Fetz, 1992; Crone et al., 1998; Courtemanche et al., 2003; Goldberg et al., 2004), but recent studies challenged this classic view by pointing out the modulation of beta oscillations during decision-making. Corticostriatal beta oscillations have been shown to be affected by decision-related variables (Leventhal et al., 2012; Spitzer and Haegens, 2017; Amemori et al., 2018; Eisinger et al., 2018), and they have been shown to encode categorical choices rather than upcoming movement (Haegens et al., 2011). In the non-human primate studies, the striatal beta oscillation was reported to be modulated during lack of dopamine (Goldberg et al., 2004), movement suppression (Courtemanche et al., 2003) and postperformance period at the end of the task execution (Feingold et al., 2015). However, the information encoded during the decision period is still unclear.

We examined features of the neural processing of valence and arousal by focusing on the unit and local field potential (LFP) oscillatory activities in the primate caudate nucleus (CN) under Ap–Av performance conditions. Studies have shown that the beta-band oscillatory signals of LFP activity recorded from the cerebral cortex (Sherlin and Congedo, 2005; Haegens et al., 2011; Schutter and Knyazev, 2012) and striatum (Leventhal et al., 2012) encode decision-variables that could vary as a function of state anxiety (Velikova et al., 2010; Poppelaars et al., 2018), depression (Saletu et al., 2010; Roh et al., 2016) and Ap-Av motivation (Amemori et al., 2018). Also for the CN neuronal units, previous work has shown that the activity of single units in the CN can encode variables related to value-based decision-making (Samejima et al., 2005; Lau and Glimcher, 2007), suggesting that the primate CN could be a functional node in affective judgment (Kim and Hikosaka, 2013; Amemori et al., 2018). In this study, we thus focused on the temporal characteristics of the CN unit and beta oscillatory activities with the aim of understanding the interaction of valence and arousal.

## Materials and Methods

### Subjects and the Ap–Av Decision-Making Task

We studied two female *Macaca mulatta* monkeys (monkey S: 7.5 kg, ∼6 years old; monkey P: 6.8 kg, ∼5 years old). We conducted the experiments under the Guide for Care and Use of Laboratory Animals of the United States National Research Council. All procedures were approved by the Committee on Animal Care of the Massachusetts Institute of Technology. We reanalyzed the same data that were published previously (Amemori et al., 2018) by newly focusing on the encoding of valence and arousal in the neural responses.

**FIGURE 1:**
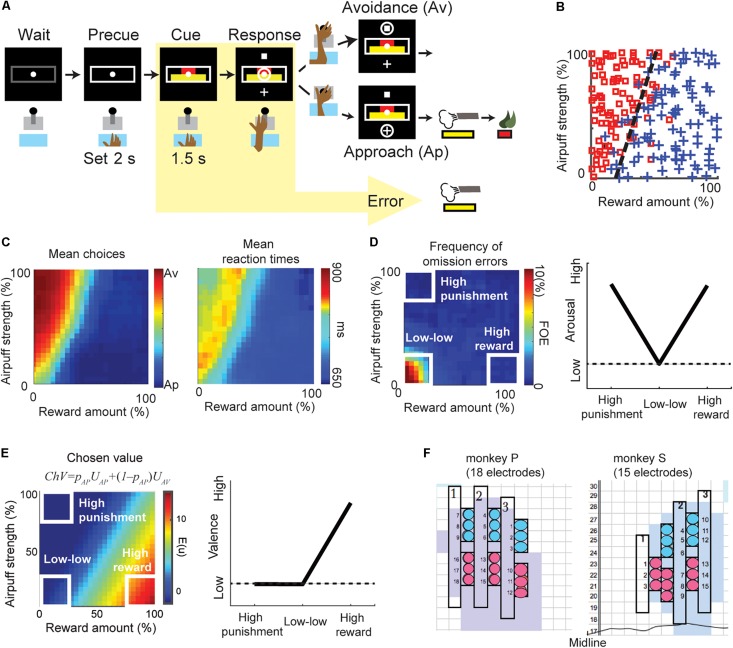
The behaviors of the Ap–Av decision-making task. **(A)** Task procedure of the Ap–Av decision-making task. During the cue period, the red and yellow horizontal bars, respectively signaling the offered amounts of reward and punishment, appeared on the monitor. The monkeys decided between acceptance and rejection of the combined offer and reported it by choosing either of two targets (cross for Ap; square for Av) that appeared during the response period. Locations of the targets were alternated randomly. When the monkey did not respond during the response period, the trial was counted as an omission error. **(B)** The Ap–Av choice pattern in a single session. The *x*-axis indicates the offered reward amount, and the *y*-axis shows the offered airpuff strength. Blue crosses indicate Ap choice. Red squares indicate Av. **(C)** Mean Ap–Av choices (left). Mean reaction times (RTs) mapped onto the decision matrix (right). Each datum was spatially smoothed by a square window (20% by 20% in the decision matrix). **(D)** Frequency of omission errors (FOE) (left) and the schematic of arousal (right). When the monkey did not move the joystick during the 3-s response period, the trial was regarded as an omission error. To compare the valence and arousal, we focused on the “high-reward,” “high-punishment,” and “low–low” condition (right). We observed omissions almost exclusively at the “low–low” condition punishment offer, suggesting that both reward and punishment facilitated task engagement. Arousal level was thus defined as a V-shape relationship as it became high either in the “high-punishment” or in the “high-reward” condition (left). **(E)** The chosen value (ChV) (left) and the schematic of valence (right). The ChV corresponds to the expected outcome value associated with the selected option. We defined valence by the ChV. Valence became high in the “high-reward” condition and low in the “low–low” condition. It became zero in the “high-punishment” condition as the monkey always chose Av in the condition. **(F)** Positions of the implanted electrodes (circles) on the recording grid system for monkey P (left) and monkey S (right). Grids were placed on the skull with 5° tilt from the horizontal plane. Electrodes were implanted in the anterior portion of the CN (light blue shading). The numbers along the midline indicate the intra-aural anterior-posterior coordinates of the grid system in millimeter. The color of the circles indicates the group of electrodes that shared the same reference signal.

Two female monkeys were trained to perform the Ap–Av decision-making task (Figure 1A) (Amemori and Graybiel, 2012). This task started when the monkey put her hand on a sensor in front of a joystick. After the 2-s precue period, a compound visual cue made up of red and yellow horizontal bars appeared in the center of a monitor in front of the monkey. The monkeys had to learn that the length of the red bar indicated the offered size of a reward, but that the length of the yellow bar indicated the offered pressure strength of an airpuff to the face that would accompany the indicated level of reward. The red and yellow bar lengths were varied independently so that during a given ∼700 trial session, many decisions could be registered for analysis. After a 1.5-s cue period, cross and square targets appeared up and down of the cue, their positions interchanging randomly from trial to trial. The monkey reported her decision during the 3-s response period by using the joystick to move a cursor to either target. When she chose the cross target (approach, Ap), we provided an airpuff with the indicated (yellow bar) pressure to the face. We then delivered the liquefied food reward with the indicated amount (red bar). When she chose the square (avoidance, Av), we did not give airpuff, but a minimal amount of reward was delivered so that we could ensure that she would continue to perform the task. If the monkey did not respond during the response period, we provided an airpuff with the indicated pressure so that she actively chose the Av choice.

### Arousal Defined by the Level of Task Engagement

Arousal and valence are both hidden variables used by psychologists to explain behavior. Their relationships to experimental observables are thus somewhat complicated. Arousal has been defined as “disposition to react with varying degrees of energy or force” (Lang et al., 1990). A lackadaisical behavioral response would thus indicate low arousal. Arousal should increase with the expectation of either reward or punishment, so anything that shows a positive correlation with either (or both) of those measures might also be taken as an indicator of arousal. To define the “arousal,” we referred to previous psychology articles (Lang et al., 1997, 1998), and examine the behavioral and physiological responses in “high-reward,” “low–low” and “high-punishment” conditions (Figure 1D, right panel). Lang et al. (1997) and Lang and Bradley (2007) produced by the international affective picture system (IAPS) by collecting pictures that could induce various pleasure and arousal responses, and then found that the relationship between the self-assessed ratings of pleasure and arousal showed a V-shape. Using this relationship, they examined psychophysiological measurements such as skin conductance, pupil diameter, and heart rate responding to each picture (Bradley et al., 2001, 2008; Lang and Bradley, 2007). They found that the physiological responses also showed a V-shape relationship and concluded that those physiological responses encoded arousal. In this article, we followed their definition of arousal and examined whether or not the unit and beta responses in the “high-reward,” “low–low,” and “high-punishment” conditions showed a V-shape.

As the monkeys made their decisions to accept or to reject the offers, depending on the offered amounts of reward and the offered strength of airpuff indicated by the cues (Figure 1B), we observed longer RTs for Av choices than for Ap choices and found that the RTs were also lengthened around the decision boundary (Figure 1C). Long RTs were thus often associated with what we imagine to be difficult choices, but in some cases, they might be taken to indicate a lack of arousal. Certain types of errors unambiguously show a lack of arousal. Error trials were classified into omission errors (i.e., failure of response during the response period) (Figure 1D) and fixation breaks during the cue period. Omission errors, by definition, represent a lack of task engagement. Lack of task engagement constitutes a disposition not to react to task events, and in the absence of a compelling distractor, it therefore equates to lack of arousal.

The frequency of omission errors (FOE) was high only when the offered reward and punishment were both low (“low–low” condition), suggesting that task engagement was enhanced by either a large reward (“high-reward” condition) or a substantial punishment (“high-punishment” condition) (Amemori et al., 2015). Because the FOE was inversely correlated with the level of task engagement, we used this feature to characterize the arousal level (Figure 1D, left). In the Ap–Av task, the FOE indeed showed V-shape relationship (Kuppens et al., 2013) by examining the values in the “high-punishment,” “low–low,” and “high-reward” conditions (Figure 1D, right), and we concluded the FOE corresponded to the level of arousal (Lang and Bradley, 2007).

### Valence Defined by Chosen Value

To infer the value judgment of the monkey, we applied an econometric model (Train, 2003; Glimcher et al., 2005). The probability of choosing the Ap choice can be written as *p*_AP_ = 1/(1 + exp(−(*U*_AP_ − *U*_AV_))). *U*_AP_ and *U*_AV_ are the utilities for choosing Ap and Av. We modeled the function *U*_AP_ − *U*_AV_ as *f*(*x*, *y*) = *ax* + *by* + *c*, where *x* was the reward size, *y* was the airpuff strength. Generalized linear regression was used to fit the function to the monkey’s choices pattern by determining the coefficients (*a*, *b*, and *c*) that minimize the mean squared error. We modeled the utility of choosing Ap as *U*_AP_ = *ax* + *by* and the utility of choosing Av as *U*_AV_ = −*c*. The chosen value – the expected outcome value associated with the selected option – was thus calculated as ChV = *p*_AP_*U*_AP_ + (1 – *p*_AP_)*U*_AV_ (Figure 1E, left). Because ChV estimates the subjective outcome value of a given offer, we defined valence as ChV. The ChV became high in the “high-reward” condition and low in the “low–low” condition. The ChV in the “high-punishment” condition is −*c*, which was close to zero, as the monkey always chose Av in the “high-punishment” condition (Figure 1E, right).

### Electrophysiological Recording of CN Units and Beta Oscillations

After placement of the recording chamber, we implanted for chronic recording sets of 18 platinum-iridium electrodes (impedance, 0.1–1.0 MΩ; FHC) targeting the CN in the left hemisphere in the monkey P, and 15 electrodes in the right hemisphere in the monkey S (Figure 1F) (Feingold et al., 2012). We simultaneously recorded spike and LFP activities while the monkeys performed the Ap-Av decision-making task. The original filter setting during recording LFP was between 0.1 Hz to 32k Hz. We recorded 958 LFP activities from different recording sites. Most of these LFPs (81%, 780 channels) exhibited beta-band (13–28 Hz) oscillations. Because over three-quarters of them (76%, 728 channels) showed peaks within the beta range, we focused on these beta oscillations. The majority of the beta oscillations (86%, 667 channels) were defined as task-related, as they exhibited significant changes in magnitude (*P* < 0.05, two-tailed *z*-test, Bonferroni corrected) during the time from the precue period (the 4-s period before cue onset) to the cue period. To reduce volume conductance, local-averaged reference signals were computed by averaging the signals from the electrodes in each local electrode group, as shown in Figure 1F.

**FIGURE 2:**
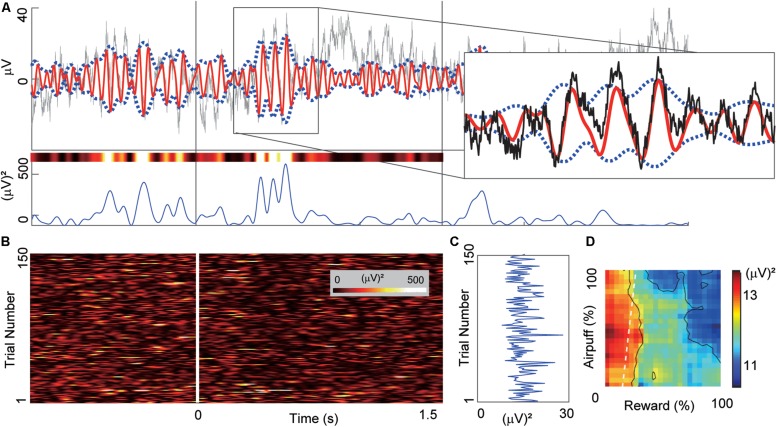
Example of beta oscillation recorded from a CN electrode. **(A)** Example of the LFP activity recorded from a CN electrode aligned to the onset of the cue period. The time scale is the same as in **(B)**. Gray and red lines in the top panel indicate the LFP activity and the band-pass-filtered (13–28 Hz) activity. We derived the power magnitude using the difference between the upper and lower envelopes that were represented by blue dotted lines. The right inset shows a magnified view of the region inside the rectangle. The bottom panel shows the power magnitude of the envelopes. The variously colored bar above the power trace shows the same power data color-coded using the same color scale as in **(B)**. **(B)** The trial-by-trial power magnitudes as a pseudo-colored raster plot (inset shows color scale). The *x*-axis indicates the time from the cue onset. *Y*-axis indicates the trial number. **(C)** The mean power of the beta magnitudes averaged over the 1.5-s cue period. **(D)** The beta response that was produced by mapping the cue-period mean power onto the decision matrix. The mapped data were spatially smoothed by a 20%-by-20% square window. *X*-axis and *Y*-axis indicate the offered sizes of reward and punishment, respectively.

Figure 2 illustrates the procedures used to calculate beta power magnitudes recorded from a single CN electrode channel. To characterize the task-related modulation of the beta responses during the cue period, we performed band-pass filtering to extract the beta power for each trial (Figures 2A,B). For each trial, we derived the beta power averaged over the cue period (Figure 2C) and referred to it as a “cue-period beta magnitude.” We projected the activities onto the decision matrix (Figure 2D). We refer to the matrix of beta power magnitudes as a “beta response.” Similarly, for the neuronal unit activities, we focused on the spike count during the cue period for each trial and referred to it as a “cue-period unit activity.” We projected the activities onto the decision matrix and referred to it as a “unit response.”

### Multidimensional Scaling and Clustering of Beta Responses and Units

**FIGURE 3:**
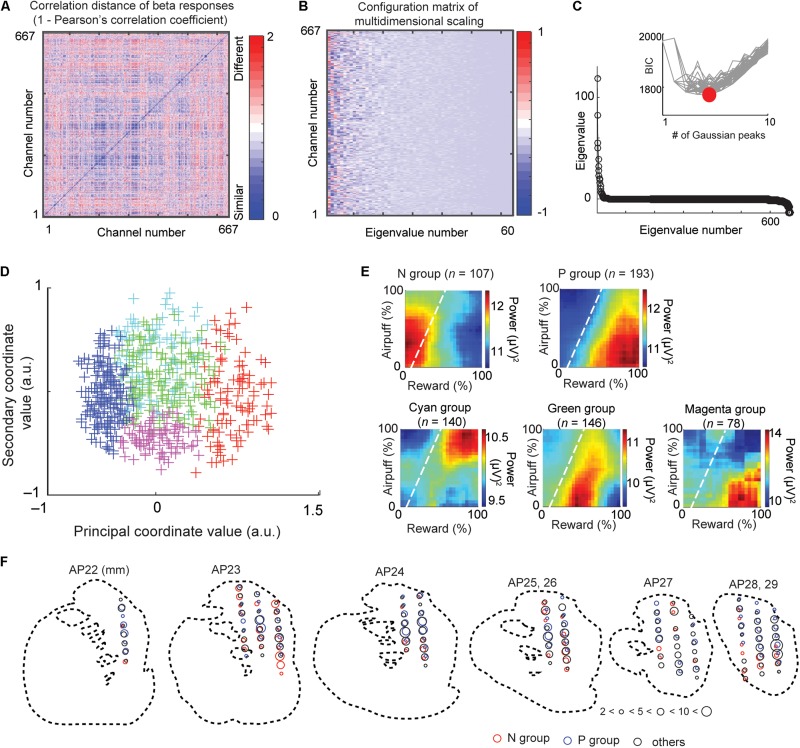
Multidimensional scaling and clustering of beta responses. **(A)** Matrix of correlation distance between pairs of all beta response matrices (*D* = [*d*_*ij*_]). The color of each element shows the correlation distance (*d*_*ij*_ = 1 - *r*_*ij*_), where *r*_*ij*_ is the cross-correlation between decision matrices of beta response *i* and response *j*. **(B)** Configuration matrix derived from the multidimensional scaling. **(C)** Eigenvalues showing the explanatory power of each feature dimension. Inset shows the BIC values for different numbers of Gaussian peaks. Gray lines indicate the BIC values for each of many independent runs of a procedure that did the mixture-of-Gaussian fitting for each number of peaks from 1 to 10. The minimum BIC was given by five Gaussian peaks and denoted as a red circle. **(D)** Beta response matrices projected onto the first two dimensions of the MDS. Each cross indicates an individual channel. The color indicates the group that the channel belongs to (red: N group, blue: P group, green, cyan, and magenta: other groups). **(E)** The group means of beta responses in the decision matrix. Each group (N, P, cyan, green, or magenta group) was defined by the MDS clustering shown in **(D)**. **(F)** Spatial distribution of sites at which we recorded LFPs classified as N (red), P (blue), and other (black) groups. The size of each circle indicates the number of LFPs at the location. Data from monkey S were projected onto outline drawings of the striatum of monkey P.

We performed following multidimensional scaling (MDS) to identify groups of beta and unit responses by the similarities of each class (beta and spike) of neural responses. This procedure does not require explanatory variables that we arbitrarily define, and the groups identified by the MDS procedure extract the features that the beta responses originally contain. Figure 3 illustrates the procedure of an unbiased clustering of the beta responses to identify the groups of recorded LFPs whose activities similarly responded to the cue. We calculated a correlation distance matrix *D* = [*d*_*ij*_] where *d*_*ij*_ = 1 – *r*_*ij*_ using the correlation *r*_*ij*_ between beta responses of channel *i* and *j* (Figure 3A). We performed MDS using the *mdscale* function of MATLAB to derive feature coordinates that maximally differentiated the responses (Figure 3B). The eigenvalue *d* (Figure 3C) represented the explanatory power of each dimension. To cluster these channels, we then fitted a Gaussian mixture distribution (using *fitgmdist* function of MATLAB) to the set of values in each dimension of the new feature space (i.e., to each column of the configuration matrix). We adopted the Bayesian information criterion (BIC) to derive the number of groups (Figure 3C). We then projected each channel onto the first two dimensions of the MDS (called MDS map) (Figures 3D–F).

### Regression Analyses for CN Beta Responses and Unit Responses

**FIGURE 4:**
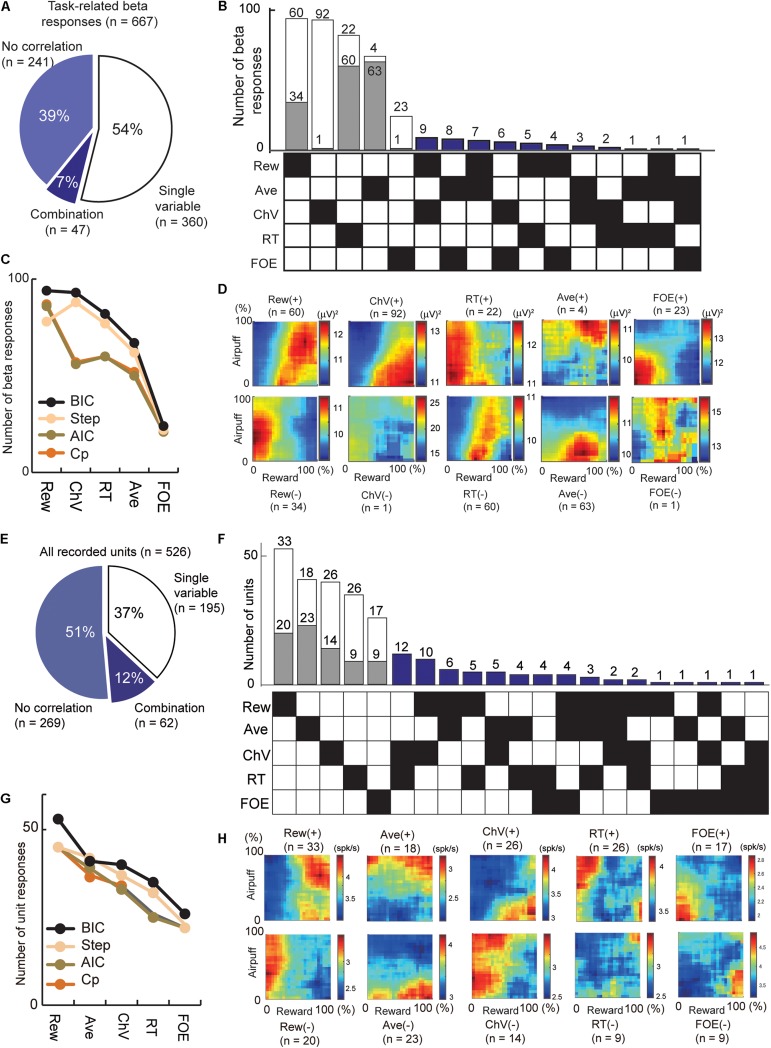
Regression analyses that extract the information encoded by the cue-period beta magnitudes and cue-period unit activities. **(A)** The number of beta responses classified by all-possible subset regression. The proportion of cue-period beta magnitudes explained by a single (54%) and combination (7%) of variables. **(B)** All-possible subset regression analysis of beta responses using the five explanatory variables (Rew, Ave, ChV, RT, and FOE), sorted in decreasing order of total number responses explained. The 360 responses explained by single variables were further separated into channels with responses that were correlated positively (white) or negatively (gray) with the variable. Forty-seven beta responses were characterized by particular combinations of variables indicated by black squares in the matrix on the bottom. **(C)** Classification of beta responses with all-possible subset regression analyses performed with different criteria (black, BIC; brown, AIC; orange, Mallow’s Cp), and stepwise regression analysis (cream). *Y*-axis is the number of beta responses where the best model was the single variable on the *x*-axis. **(D)** The population activity of the beta responses explained by single variables, sorted as in **(B)**. Those correlated positively (+) and negatively (−) with the variables were separately categorized. **(E)** The number of unit activities classified by all-possible subset regression. The proportion of the cue-period unit activities explained by a single (37%) and combination (12%) of variables. **(F)** All-possible subset regression analysis of unit responses using the five explanatory variables (Rew, Ave, ChV, RT, and FOE), sorted in decreasing order of total number responses explained. The cue-period unit activities of 195 units were explained by single variables and were further separated into channels with responses that were correlated positively (white) or negatively (gray) with the variable. The activities of 62 units were characterized by particular combinations of variables indicated by black squares in the matrix on the bottom. **(G)** Classification of units with different criteria as in **(C)** (black, BIC; brown, AIC; orange, Mallow’s Cp; stepwise regression analyses, cream). **(H)** The population activity of the unit responses explained by single variables, sorted as in **(F)**. Those correlated positively (+) and negatively (−) with the variables were separately categorized.

To examine the features encoded by the cue-period beta responses and unit activity (Figure 4), we applied all-possible subset regression analysis with five selected explanatory variables, consisting of offered reward size (Rew), offered airpuff size (Ave), ChV (Figure 1E), RT (Figure 1C) and FOE (Figure 1D). Linear regression analyses were performed exhaustively using every possible combination of the five explanatory variables. Among the combinations of variables that explained the cue-period activity significantly well (*P* < 0.05, *F*-test of the overall fit), the combination that produced the highest BIC score was selected. We scored the quality of fit using the BIC and counted the number of channels that were best explained by a single variable or a combination of variables. We also used Akaike Information Criteria (AIC), Mallow’s Cp (Cp), and stepwise regression for the scoring (Figures 4C,G). The beta responses and unit activities used in these analyses did not have multicollinearity problems diagnosed by Belsley’s criteria (Belsley et al., 1980). We use the term “encode” to indicate that we interpreted that the unit or beta activities exhibited differential responses specifically to the variable. However, as the explanatory variables were arbitrarily introduced, it did not mean that the unit or beta exhibited selective responses only to the variables.

## Results

### Classification of CN Beta and Units

We applied regression analyses to extract the information encoded by the beta responses and unit activities (Figure 4). First, we analyzed the cue-period beta magnitudes of 667 task-related beta responses. Among them, 360 beta responses (54% = 360/667) were accounted for by one of those variables (Figure 4A). Among them, 93 beta responses (24.8%) encoded ChV and 24 responses (6.7%) encoded FOE (Figures 4B,D). The beta responses encoding ChV were regarded as those encoding valence, and those encoding FOE were regarded as arousal-encoding. We used BIC to derive these, but we confirmed that AIC, Cp, and stepwise regression provided similar results (Figure 4C).

Secondly, we examined information encoded by cue-period unit activity. We performed all-possible subset regression based on the cue-period activities of 526 CN units. We found 195 units with activities encoding single explanatory variables and did not encounter multicollinearity problems in this analysis (Figure 4E). Among them, the activities of 40 units (20.5%) encoded ChV and those of 51 units (27.6%) encoded FOE (Figures 4F,H). We used BIC to derive these but confirmed that the AIC, Cp, and stepwise regression provided similar results (Figure 4G).

### Comparing Beta and Unit That Encoded Valence and Arousal

**FIGURE 5:**
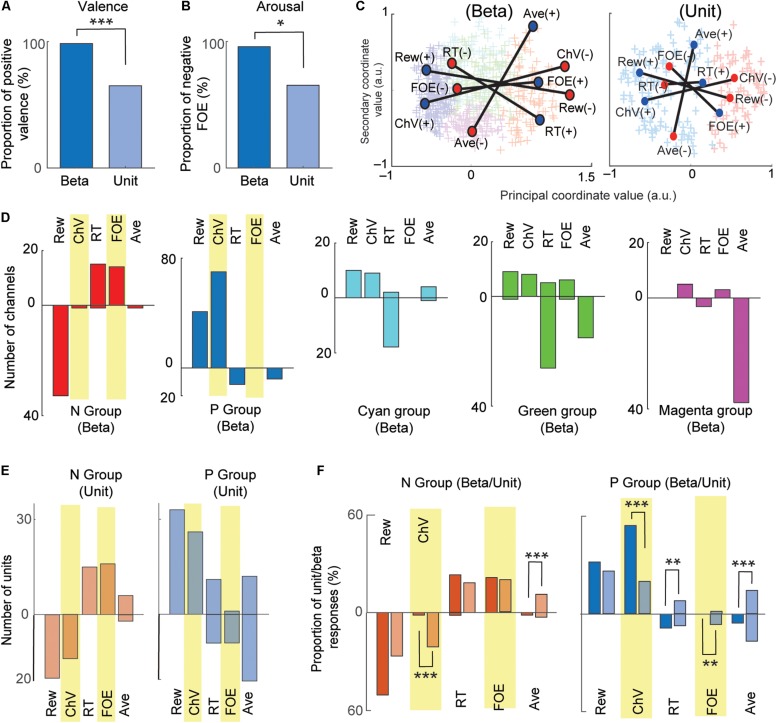
Comparison between beta and unit that encoded valence and arousal. **(A)** Comparison between valence-encoding beta and valence-encoding unit responses. The percentage of positive-valence beta responses was significantly larger than that of unit responses (Fisher’s exact test, ***P < 0.001). **(B)** Comparison between the arousal-encoding beta and unit responses. The percentage of the negative-arousal beta responses was significantly larger than that of unit responses (Fisher’s exact test, *P < 0.05). **(C)** MDS clustering for the beta (left) and unit (right) responses. The beta and unit responses were projected onto the first two dimensions of the MDS (MDS map). Each cross indicates an individual response. Two groups (P and N groups) were defined by the Gaussian mixture model. The positions of the five explanatory variables were projected onto the MDS map. Blue and red circles indicate, respectively, positive and negative correlation with the variables. **(D)** The representation of beta responses for each group identified by the MDS clustering. The number of beta responses encoding the five behavioral variables shown separately for each group. The stacked bars that go up indicated positive correlations, and those that go down indicated negative correlations. **(E)** The representation of unit responses for N and P groups identified by the MDS clustering. Stacked bars that go up and down indicate positive and negative correlations, respectively. **(F)** Comparison of beta and unit responses in the P and N groups (dark colors, beta; light colors, units). Stacked bars that go up and down indicate positive and negative correlations, respectively. Statistically significant differences between the proportion of units and proportion of beta are marked (Fisher’s exact test, ***P < 0.001, **P < 0.01).

We then compared the beta responses and unit activities that encoded valence. Among 93 channels that encoded ChV, 98.9% of beta responses (92/93) were positively correlated with ChV, putative valence (Figure 4B). By contrast, among 40 single units encoding ChV, the activities of only 65.0% (26/40) were positively correlated with the ChV, and 35.0% (14/40) showed a negative correlation (Figure 4F). Thus, among the channels that encoded valence, the proportion of beta responses encoding positive valence (98.4%) was significantly larger than that of unit responses encoding positive valence (65.0%) (*P* = 10^–5^ < 0.001, Fisher’s exact test) (Figure 5A). These results suggest that the CN beta-band oscillatory responses exhibited almost entirely selective representation for positive valence, whereas the CN unit responses represented valence both positively and negatively.

We examined arousal representation by focusing on the beta responses and unit activities that encoded the FOE. Twenty-four beta responses encoded FOE (Figure 4B). Among them, the responses of 95.8% (23/24) were positively correlated with FOE, and only one beta response was negatively correlated. On the other hand, the activity of 26 units encoded FOE (Figure 4F) either positively (65.3% = 17/26) or negatively (34.6% = 9/26). Thus, among channels that encoded arousal, the proportion of the beta responses that encoded negative arousal (i.e., positive FOE, 95.8%) was significantly larger than that of unit activities (65.3%) (*P* = 0.011 < 0.05, Fisher’s exact test) (Figure 5B). These results suggest that the arousal-encoding beta responses primarily represented low arousal by encoding FOE(+), but the arousal-encoding units responded to either of the low and high arousal conditions.

### Unbiased MDS Clustering of Beta and Unit Responses

The above decoding procedure depends on explanatory variables that we arbitrarily defined. It is still essential to classify recordings only by their response features without introducing any arbitrarily defined variables. We thus introduced the MDS clustering procedure (Figure 3) and identified groups of beta and unit responses only by their similarities (Figure 5C). The MDS clustering of the beta responses yielded five groups, and those of the unit responses yielded two groups. Both for beta and for the unit responses, we focused on the N and P groups of the beta and unit responses because they were, respectively, located at the minimal and maximum principal coordinate values. To interpret the meaning of the principal coordinates, we projected the explanatory variables onto the MDS feature space by transforming each of the population response matrices from Figures 4D,H into the MDS feature space. Each explanatory variable has a response map for positively correlated responses and one for negatively correlated responses, so that each explanatory variable corresponds, in Figure 4, to a pair of matrices that map to a pair of points in the MDS feature space. Each such pair of points is plotted with a line connecting them in Figure 5C. We found that the principal coordinates of the MDS feature spaces for both beta (Figure 5C, left) and unit (Figure 5C, right) responses similarly represent the differential activities related to the offered reward (Rew) and the chosen value (ChV).

To cluster the beta responses, the MDS identified a group of beta responses (the “N group”) that mainly coded negatively for the offered reward size (33/65 channels, Figure 5D). The population activity of N-group beta responses (Figure 3E) showed peak activation near the “low–low” condition, raising the possibility that the N-group beta responses could primarily be activated for lack of arousal. In fact, this group contained a large number of channels that positively coded for either RT (15/65), which can also be interpreted as reflecting lack of arousal, or for FOE (14/65), which unambiguously indicates lack of arousal. Only two channels correlated with other explanatory variables, only one of which was ChV (valence). The N group of beta responses was thus consistently associated with an apparent lack of interest or motivation to perform the task. By contrast, the N group of unit responses (Figure 5E) was much more heterogeneous, including both positive and negative responses related to offered aversion, as well as negative coding for ChV. Among 129 P-group of beta responses, 70 responses (54.2% = 70/129) encoded ChV(+), but none of the P group encoded FOE (Figure 5D). These results suggest that the subsets of P-group beta responses encoded valence. None of them explicitly encoded arousal (as FOE), and only 8.5% (11/129) encoded RT. The P-group units, like the N-group units, responded in relation to multiple features, not exclusively in relation to valence (Figure 5E).

To clarify these relationships, we directly compared the proportions of the beta and unit responses that encoded each variable in each group (Figure 5F). The proportion of valence-encoding unit responses in the N group (15/71) was much larger than that of the beta responses in the N group (1/65) (*P* = 0.0011 < 0.01, Fisher’s exact test). These results suggest that, while the N-group units encoded valence and arousal almost equally in proportion, the N-group beta responses did not encode valence. The proportion of arousal-encoding unit responses in the P group (10/126) was also significantly larger than that of the beta responses (0/129) (*P* = 0.0017 < 0.01, Fisher’s exact test), whereas that of valence-encoding beta responses in the P group (70/129) was significantly larger than that of the unit responses (25/126) (*P* = 10^–4^ < 0.001, Fisher’s exact test). These results suggest that, while substantial proportions of P-group units encoded, respectively, both valence and arousal, the majority of P-group beta responses encoded positive valence only, not arousal.

Using MDS clustering, we identified five distinct groups that showed differential decision-related features without introducing arbitrarily defined explanatory variables. The mean responses identified by the MDS clustering (Figure 3E) thus reflected the actual contours across the decision matrix of the beta responses within each cluster. After the clustering, we performed regression analysis on each cluster to interpret its decision-related features. We focused on the N and P groups in both the beta and unit responses because the principal coordinate value was sufficient to distinguish them in the MDS map. Unit response clusters related to a wide variety of different decision-related features, whereas the P and N groups of beta responses related to distinctive decision-related features. The N group beta responses encoded FOE (our marker for arousal) but not ChV (our marker for valence), suggesting that they mainly represented negative arousal. The fact that negative offered reward size (Rew) was the only other feature encoded by a substantial number of N group beta channels is consistent with this interpretation. By contrast, most of the P group beta responses encoded ChV but not FOE, suggesting that the P group mainly represented positive valence, which is consistent with the fact that almost all of the other channels in the P group encoded positive offered reward size. These results suggest that the beta responses mainly exhibited selective activation for the high-valence and low-arousal conditions, whereas the unit activities responded to various decision-related features in the Ap–Av decision-making.

### Unit Representation of Arousal and Valence

**FIGURE 6:**
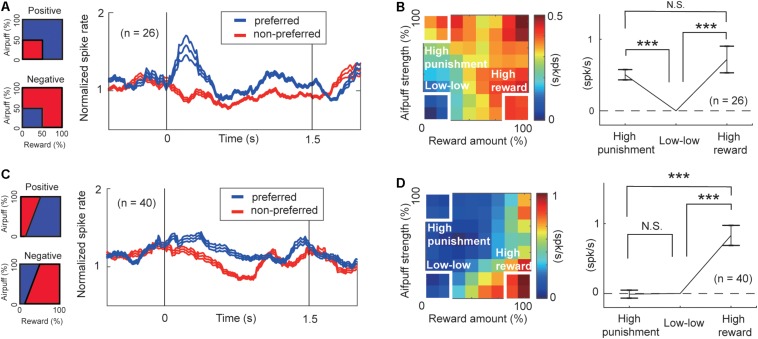
Features of units encoding arousal and valence. **(A)** Population activity of the arousal-encoding units for the preferred (blue) and non-preferred (red) conditions. The time courses of the activities of the FOE unit were normalized by their precue period activities. Preferred and non-preferred conditions of arousal-positive or FOE(−) unit (top) and of arousal-negative or FOE(+) units (bottom) are shown on the left. **(B)** Mean of the differential activity relative to the “low–low” condition. Activities of FOE(−) units and the inverse of FOE(+) activities were mapped on the decision matrix (left). We compared the population mean activity in the “high-punishment,” “low–low,” and “high-reward” conditions (Paired *t*-test, ***P < 0.001; N.S.: *P* = 0.29 > 0.05). The arousal-encoding units did not discriminate between high-punishment and high-reward conditions. **(C)** Population activity of valence-encoding units. Preferred (blue) and non-preferred (red) conditions of the ChV(+) unit (top) and of ChV(−) units (bottom) are shown on the left. **(D)** Mean of the differential activity relative to the “low–low” condition. Activities of ChV(+) units and the inverse of ChV(−) activities were mapped on the decision matrix (left). We compared the population mean activity in the “high-punishment,” “low-low,” and “high-reward” conditions (Paired *t*-test, ***P < 0.001; N.S.: *P* = 0.87 > 0.05).

To characterize the response features of the arousal-encoding units, we focused on the activities of FOE units in the “low–low,” “high-reward,” and “high-punishment” conditions. We selected these conditions so that we can compare our neural results with the definition of arousal level proposed by previous studies (Lang and Bradley, 2007; Kuppens et al., 2013). The activities of FOE(+) and those of FOE(−) units exhibited, respectively, positive and negative correlations with FOE. Regardless of the sign of the correlation with the FOE, both of them could be involved in the arousal process. We thus flipped the sign of the FOE(+) activity to align to show the strength of the relationship with arousal consistently across the FOE(+) and FOE(−) groups. For each unit, we defined the preferred condition as the one in which the activity was higher than that in the other conditions (Figure 6A). The preferred condition of the FOE(+) units, in which they exhibited higher activity, was the “low–low” condition. The FOE(−) units preferred conditions other than the “low-low” condition. The time course of the population activities of the arousal-encoding units exhibited significant differences during the early stage of the cue-period.

Further, we compared the population activities of arousal-encoding units in the “high-reward” and “high-punishment” conditions to confirm that these activities were not influenced by valence. The primary feature of the arousal-encoding units is that they did not discriminate between high-punishment and high-reward conditions. We compared the means of the increases in activity from activity in the “low–low” condition (Figure 6B, right). In this figure, the sign of the FOE(+) activities is inverted to allow comparisons of the mean magnitudes of the correlations with the arousal level. We observed a significant increase either in the “high-reward” condition (*P* = 10^–3^ < 0.001, paired *t*-test) or in the “high-punishment” condition (Figure 6B) (*P* = 10^–8^ < 0.001, paired *t*-test). There was no significant difference between the means in the “high-reward” and “high-punishment” conditions (*P* = 0.29 > 0.05, paired *t*-test), indicating that the arousal-encoding units responded to the strength of the offer rather than to its value. As these units did not discriminate reward from punishment, we concluded that the FOE units encoded arousal exclusively and were not influenced by valence, at least in their mean population activity.

To examine the features of valence-encoding units, we focused on ChV units. We defined the preferred condition of ChV(+) units to be the condition in which the monkey made Ap choices, and the preferred condition of the ChV(−) units to be the condition in which the monkey made Av choices (Figure 6C). We mapped the activities of ChV(+) units and the inverted activities of ChV(−) units onto the decision matrix shown in Figure 6D. We calculated the means of the increased activities from the “low–low” condition. We then observed a significant increase in the “high-reward” condition (*P* = 10^–6^ < 0.001, paired t-test) but no change in the “high-punishment” condition (*P* = 0.87 > 0.05, paired *t*-test), indicating that activities of the valence-encoding units dissociated reward and punishment.

These findings suggest that during the performance of the Ap–Av task, the CN units that we sampled could be divided into units responding in relation either to arousal or to valence. The arousal-encoding units were activated only for the magnitude of the offer without discriminating between the levels of reward and punishment indicated by the offers. The value-encoding units represented the value of the expected outcome, in having differential activities for the offered levels of reward and punishment.

### Beta Representation of Valence and Arousal

**FIGURE 7:**
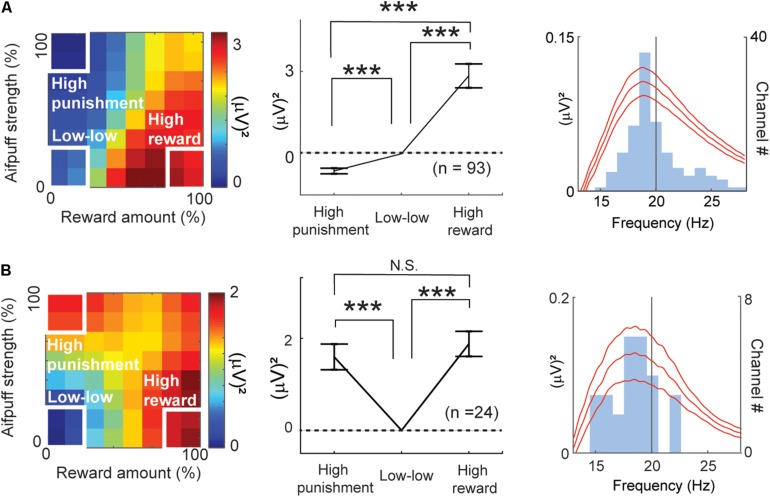
Features of beta responses encoding valence and arousal. **(A)** Features of valence-encoding beta responses. The left panel shows the mean of the differential activity from the “low–low” condition for the valence-encoding beta responses. Activities of valence-encoding beta responses were mapped onto the decision matrix. Those of ChV(+) beta responses were added, and those of ChV(−) were subtracted. The middle panel shows the mean increase in activities from the “low-low” condition. The valence-encoding beta responses discriminated the “high-punishment” and “high-reward” conditions (Paired *t*-test, ***P < 0.001), showing differential responses to reward and punishment. The right panel shows the mean (±SEM) power spectra of the valence-encoding beta responses (baseline-subtracted), peaking at 18.7 Hz. The light blue histogram indicates the distribution of peaks of power spectra. **(B)** Features of arousal-encoding beta responses. The left panel shows the mean of the differential activity from the “low–low” condition for arousal-encoding beta responses. Activities of FOE(−) beta responses were added, and those of FOE(+) were subtracted. The middle panel shows the mean increase in activities from the “low–low” conditions. The activities in the “high-punishment” and “high-reward” were significantly higher than those in the “low–low” condition (Paired *t*-test, ***P < 0.001). The arousal-encoding beta responses did not discriminate the “high-punishment” and “high-reward” conditions (N.S.: *P* = 0.47 > 0.05). The right panel shows the mean (±SEM) power spectra of the arousal-encoding beta responses (baseline-subtracted), peaking at 18.9 Hz. The light blue histogram indicates the distribution of peaks of power spectra.

By all-possible subset regression, 93 beta responses were classified as valence-encoding. Among them, 92 responses showed a positive correlation with the ChV [ChV(+)]. These are mapped onto the decision matrix in Figure 7A. Only one showed a negative correlation with the ChV [ChV(−)], and the inverse of its activity is mapped onto the same decision matrix. We calculated the mean of the differential activities relative to the “low–low” condition. The mean was significantly lower in the “high-punishment” condition (*P* = 10^–8^ < 0.001, paired *t*-test) and was significantly higher in the “high-reward” condition (*P* = 10^–7^ < 0.001, paired *t*-test), suggesting that the valence-coding beta responses discriminated reward from punishment.

The regression analyses identified 24 beta responses as encoding arousal (Figure 4A). The beta responses encoding FOE(−) and the inverses of the beta responses encoding FOE(+) were mapped onto the decision matrix (Figure 7B). We again calculated the mean of the differential activities from the “low–low” condition. The mean exhibited a significant increase in both the “high-punishment” (*P* = 10^–4^ < 0.001, paired *t*-test) and “high-reward” conditions (*P* = 10^–6^ < 0.001, paired *t*-test). However, the mean activities were not different between “high-reward” and “high-punishment” conditions (*P* = 0.47 > 0.05, paired *t*-test). These results suggest that the arousal-encoding beta oscillation responded to the magnitude of the offer without discriminating between reward and punishment during the decision period. As the beta oscillation did not discriminate reward from punishment, we concluded that the FOE beta responses exhibited exclusive responses to arousal and were not influenced by valence. The peak frequencies of most of the valence-encoding (Figure 7A) and most of the arousal-encoding beta responses (Figure 7B) were less than 20 Hz, within the low-beta range (13–20 Hz).

### Temporal Profiles of Beta Responses Encoding Valence and Arousal

We analyzed the timing at which discrimination of upcoming choices could be detected by performing cumulative onset analyses. We calculated the beta power magnitude at each time point, after smoothing with a moving average filter (window = 100 ms). We defined discrimination onset as the earliest time at which the test consecutively returned the required significance level (*P* < 0.05, Wilcoxon rank-sum test) for 100 ms.

**FIGURE 8:**
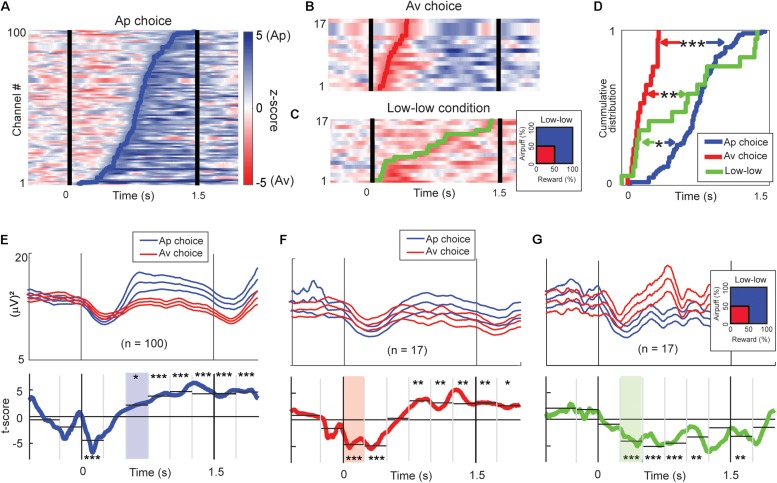
Time-course of beta responses encoding valence and arousal. **(A)** The time-course of Ap–Av discrimination ability for each Ap-responding beta channel represented by *z*-score of the Wilcoxon rank-sum test. The *z*-scores are shown as pseudocolor rasters, with shades of blue indicating higher power for Ap choice, and red indicating higher power for Av choice. We defined the onset of choice discrimination as the earliest time at which the test returned *P* < 0.05 consecutively for more than 100 ms. The blue line indicates the onset of the increase of the beta magnitude for the Ap choice. **(B)** The time-course of Ap–Av discrimination ability for each Av-responding beta channel, as in **(A)**. The red line indicates the onset of the increase of the beta magnitude for the Ap choice. **(C)** The time-course of discrimination ability for different arousal conditions. The *z*-scores are shown with shades of blue indicating higher power for high arousal conditions, and red indicating higher power for the “low–low” condition. The green line indicates the onset of the increase of the beta magnitude for the “low–low” condition. **(D)** The cumulative onset times at which beta responses discriminated between upcoming Ap and Av choices or between high and low arousal levels. The onsets of increase in the magnitude for the Av choice (red line) were significantly earlier than those for the “low–low” conditions (green line; **P < 0.01, Kolmogorov–Smirnov test) and for the Ap (blue line) choice (***P < 0.001). The onsets of increase for the “low–low” conditions were significantly earlier than those for the Ap choice (*P < 0.05). **(E)** Means (±SEM) of the beta power time course of the Ap-responding channels (top; blue traces = Ap, red traces = Av). Two-sided *t*-tests were performed for the time points to show the *t*-scores (bottom, blue line) of the differential activity between Ap and Av choices. We aggregated the activities into 250-ms bins to derive the significance level of the discrimination (*P < 0.05, **P < 0.01, ***P < 0.001, two-sample *t*-test). The light blue shows the first bin that showed a significant increase in the Ap condition. **(F)** Means (±SEM) of the beta power time course of the Av-responding channels (top; blue traces = Ap, red traces = Av). We also show the *t*-scores (bottom, red line) of the differential activity between Ap and Av choices (bottom). The light red indicates the first bin first, which showed a significant increase in the Av condition. **(G)** The group means (±SEM) of the beta power time course of the channels that showed an increase for the “low–low” condition (top; red traces = “low–low”). We also show the *t*-scores (green line) of the differential activity for the “low–low” and other conditions (bottom). The light green indicates the first bin that showed a significant increase in the “low–low” condition.

One hundred beta responses exhibited differential activities with higher magnitude for Ap choices with onsets in the latter phase of the cue period (Figure 8A). Among all task-related beta responses, 17 beta responses showed higher activities for Av choices, and their discrimination onsets were in the early stage of the cue period (Figure 8B). We also analyzed the timing of discrimination between the “low–low” and the other conditions (Figure 8C). Seventeen beta responses exhibited a higher magnitude for the “low–low” condition. By examining the cumulative distributions of the onset times, we found that different classes of beta responses were sequentially activated, first for the Av choice (or low-valence), then for the “low–low” condition (or low-arousal), and finally for the Ap choice (or high-valence) (Figure 8D).

We examined the temporal patterns of the differential activity by separating the 1.5-s cue period into six 250-ms bins. The population activity of the 100 beta responses that had higher activity magnitudes for the Ap choice exhibited higher rebound 500 ms after the cue onset and initial shallow dip in both (Figure 8E). The population activity of the 17 beta responses that showed higher magnitude for the Av choice exhibited significantly smaller suppression for the Av choice immediately after the cue onset (Figure 8F). The population activity of the 17 beta responses encoding low arousal (Figure 8G) exhibited a significant difference in the second 250-ms bin. These results agree with the analysis of the cumulative distributions of onset times in indicating the sequence of beta response during the decision period was the Av choice first, then the “low–low” condition, and the Ap choice last.

## Discussion

A classic view is that beta oscillations in decision-making reflect motor preparation. However, recent studies have pointed to the direct modulation of beta oscillations during decision-making (Haegens et al., 2011; Leventhal et al., 2012; Spitzer and Haegens, 2017; Amemori et al., 2018; Eisinger et al., 2018). Also, for the CN unit activities, unit activities have been found to exhibit correlations with the chosen value (Samejima et al., 2005; Lau and Glimcher, 2007, 2008; Yamada et al., 2007, 2013) and aversive stimuli (Blazquez et al., 2002). Further work has provided evidence that the primate CN is causally involved in associative learning (Williams and Eskandar, 2006; Kim and Hikosaka, 2013; Santacruz et al., 2017), hyperactive behavior (Worbe et al., 2011), and Ap–Av decision-making (Amemori et al., 2018; Saga et al., 2019). These studies suggest that the primate CN is a node in neural circuits that determine the Ap–Av decision, and that beta oscillations could reflect activity states underlying such choice behavior. Psychological theories suggest that task engagement or motivation to perform the task is necessary for Ap–Av decisions (Lazarus, 1991; Ben-Eliyahu et al., 2018). Cortical regions that could specifically be involved in task engagement rather than affective judgments have been identified (Roesch and Olson, 2004; Northoff et al., 2006). Here we examined neuronal processes in the CN related to Ap–Av decision-making by focusing on activities correlated with valence and arousal.

### CN Unit Activity and Beta-Band Oscillatory Activity Are Differentially Related to Valence and Arousal

We identified dissociable groups of CN beta-band responses and unit activities encoding valence and arousal. The key to this distinction rests in the task structure that required the monkeys to integrate reward and punishment. The expected outcome value of the combined offers, here represented as chosen value (ChV), was increased by the offered reward but was decreased by the offered punishment. By performing regression analyses, we found a distinctive group of unit and beta oscillatory activities that specifically encoded the ChV. Their activities increased in the “high-reward” condition compared to those in the “high-punishment” and “low–low” conditions.

One of the distinctive features of arousal is that it does not obligatorily depend on a distinction between the positive and negative aspects of the offers (Bradley et al., 2008; Kang et al., 2014). Here, we identified groups of CN unit and beta oscillatory activities that preferentially occurred during the “low–low” conditions, here identified by performance omissions (FOE), and their activities did not discriminate between the levels of forthcoming reward and punishment indicated by the offers. For unit and beta responses encoding FOE, we compared the means of the differential activities relative to the low arousal “low–low” condition and confirmed that there was no significant difference between the means in the “high-reward” and “high-punishment” conditions (Figures 6B, 7B). We thus conclude that the CN contains both arousal-related units and beta oscillatory activity related to the strength of the offers irrespective of their value.

### Differences in the Number of Units and Beta Responses Encoding Valence and Arousal

We found sharp differences in the numbers of the recorded unit and beta oscillatory activities that encoded ChV and FOE as calculated with the same regression analyses. The unit activities exhibited either a positive or a negative correlation with the ChV, whereas the beta-band responses nearly exclusively had a positive correlation with the ChV. The unit activities had either positive or negative correlations with the FOE, but the beta responses nearly exclusively had a positive correlation with the FOE. The difference in the number of these units and beta responses suggests differential functions of units and beta oscillation, with the beta responses mainly exhibiting differential activity for the high-valence and low-arousal conditions, and the unit activities responding in relation to valence as well as other features of the Ap-Av decision-making.

The above regression analyses require arbitrarily determined explanatory variables. We thus also performed unbiased (MDS) clustering to extract the groups of LFP recordings in which the beta responses exhibited similar patterns when mapped onto the behavioral decision matrix (Figures 3D,E). The MDS clustering identified two major distinctive groups of beta responses that we named the P and N groups. Notably, the P group of beta responses contained valence-encoding responses but did not contain arousal-encoding responses at all. By contrast, the N group of beta responses contained arousal-encoding responses but contained very few valence-encoding responses (Figure 5D). These results further support our hypothesis that the P and N groups reflect neural processes related to valence and arousal.

### Prospective Coding of Upcoming Target

In this study, the Ap and Av choices were associated with cross and square targets. Neural responses that were categorized as those encoding upcoming Ap and Av could thus represent prospective images of the targets. In a type of matching to sample task, in which the monkeys needed to recall the images of pictures, researchers reported that some neurons exhibited prospective coding of an object (Sakai and Miyashita, 1991; Rainer et al., 1999). In our current paradigm, although the monkeys were not required to recall the visual target object to be chosen, we could not entirely exclude the possibility that the Ap and Av neurons contained information of the object to be selected. However, the neural responses of valence (Figures 6C,D) exhibited parametric modulation representing the expected value of the outcome (Watanabe, 1996) rather than the discrete visual target identities. It is thus unlikely that our valence-encoding neural responses are only prospectively coding the visual target objects.

We also note that the monkeys did not know which way they would have to move following the decision period because the locations of the cross and square targets were varied randomly from trial to trial. Thus responses during the decision period might be related to aspects of readiness to respond, for example, but not to the direction of forthcoming joystick responses.

### Sequential Beta Responses to Valence and Arousal

Lastly, we examined the timing of encoding valence and arousal. The LFP is considered as a summation signal of excitatory and inhibitory membrane currents from neurons around the recording site (Buzsáki et al., 2012; Einevoll et al., 2013) and perhaps to glial activity (Tewari and Parpura, 2015). The onset analyses demonstrated that the modulation of the beta responses was sequential, according to condition (Figure 8). Therefore, the sequential encoding of the population of the beta responses could reflect the summation of the unit activities dominating at each temporal stage. The time-course of these beta responses showed that the beta oscillations exhibited initial “suppression” and later “rebound” in power during the Ap–Av decision-making (Figures 8E–G). The initial “suppression” differentially responded in relation to the value exhibiting shallower “suppression” for low-value conditions (Figure 8F). The “rebound” activity exhibited a high magnitude for the low-arousal condition (Figure 8G), followed by a high “rebound” activity for the Ap choice (Figure 8E). Previous studies have reported that the beta oscillations in the sensorimotor cortex show a marked decrease in power and assumed it to reflect local desynchronization (Pfurtscheller and Lopes da Silva, 1999). The beta “suppression” was often followed by a “rebound” of power, which is assumed to be resynchronization after movement (Pfurtscheller et al., 1996; Pfurtscheller and Lopes da Silva, 1999; Kilavik et al., 2013). Importantly, at the timing of the initial “suppression” of the beta responses, the neuronal unit activities exhibited an increase in activity, suggesting that the task features encoded by units and beta magnitudes exhibit opposite response patterns, which was also observed in our previous analysis (Amemori et al., 2018). Here we report that such a sequential pattern of beta desynchronization and resynchronization could also be part of the underlying mechanism of Ap–Av decision-making. Increased levels of the cholinergic drive are assumed to underlie aberrant striatal beta oscillations (McCarthy et al., 2011). That pattern of beta activity reported here could be a reflection of the activity of striatal cholinergic interneurons, and the desynchronization triggered by the visual cue could be additionally enhanced by the arrival of synchronous signals from the cerebral cortices (Crone et al., 1998). From the dorsolateral prefrontal cortex and the ACC, we had recorded neuronal activity related to valence and arousal during the task period when the CN beta oscillation exhibited desynchronization in the present data set (Amemori et al., 2015). It is thus possible that the CN beta oscillation could be involved in integrating valence and arousal to allow a decision of whether to engage in the task or not during the early phase of the cue period. After the monkey decided to engage in the task, the integrated values represented by the N group beta oscillation could be transferred to the P group beta oscillation that explicitly encoded the upcoming choice variables.

Beta-band oscillatory activity has also been implicated in the maintenance of the current status (Engel and Fries, 2010), and the N group beta responses could be related to this function. In our previous study (Amemori et al., 2018), we found that microstimulation of the CN could induce abnormally repetitive Av choices. By the effective CN microstimulation, we observed a significant enhancement of the beta magnitude before the presentation of the next offers following Av choices. Such a precue beta oscillation may have maintained a continuous negative state after Av choices and might have played a role in the linkage between ongoing emotional status and the upcoming decision. In this study, we found that N group beta responses represented negative arousal in the early phase of the decision period, suggesting a possible link with the precue beta oscillation. Since the N group beta activity appeared early in the decision period, it could contribute to the linkage between the prior emotional state and the current decision process. However, further study is needed to prove this hypothesis. The key findings of this study are that there are distinctive decision-related features of the beta-band oscillations and unit responses that we observed in the CN during Ap–Av decision-making and that the distinctive timing of the beta responses are condition-dependent. First, by taking advantage of the fact that reward and punishment differentially influence valence and arousal in Ap–Av decision-making, we tested for, and found, dissociable groups of CN beta responses and unit activities encoding valence and arousal. Whereas the population of CN units from which we recorded contained units with valence activities of both polarities, the beta responses were almost entirely positively correlated with FOE and ChV. Therefore, the beta responses mainly exhibited exclusive activation for the high-valence and low-arousal conditions, whereas the unit activities responded for positive and negative valence, as well as other features of the Ap–Av decision-making. Secondly, by examining the onset of the discrimination of conditions exhibited by the beta-band responses, we found that the beta responses were sequentially activated for strongly negative-valence, for low-arousal, and for strongly high-valence conditions. If the beta-band oscillations reflect the sum of these neuronal activities, then the sequential changes in beta representation might reflect the temporal shift in the priority of CN information processing. Initially, the CN activity might link to an emotional response to the offer, and then to a decision to engage in the task, and then to the final choice of a behavioral plan to obtain the best possible final value.

## Data Availability Statement

All datasets generated for this study are available upon request.

## Ethics Statement

The animal study was reviewed and approved by The Committee on Animal Care of the Massachusetts Institute of Technology.

## Author Contributions

KA, SA, and AG designed the experiments and performed the surgeries. KA and SA collected the data. KA and DG analyzed the data. KA wrote the manuscript. All authors edited the manuscript.

## Conflict of Interest

The authors declare that the research was conducted in the absence of any commercial or financial relationships that could be construed as a potential conflict of interest.
